# Bibliometric Analysis of Network Pharmacology in Traditional Chinese Medicine

**DOI:** 10.1155/2022/1583773

**Published:** 2022-06-15

**Authors:** Runpei Miao, Qinggang Meng, Chennan Wang, Wenjing Yuan

**Affiliations:** School of Traditional Chinese Medicine, Beijing University of Chinese Medicine, Beijing 100029, China

## Abstract

**Aim:**

We evaluated the developmental process, research status, and existing challenges of network pharmacology. Moreover, we elucidated the corresponding solutions to improve and develop network pharmacology.

**Methods:**

Research data for the current study were retrieved from the Web of Science. The developmental process of network pharmacology was analyzed using HisCite, whereas cooccurrence analysis of countries, institutions, keywords, and references in literature was conducted using CiteSpace.

**Results:**

In literature, there was a trend of annual increase of studies on network pharmacology and China was found to be the country with the most published literature on network pharmacology. The main publishing research institutions were universities of traditional Chinese medicine (TCM). The keywords with more research frequency were TCM, mechanisms, molecular docking, and quercetin, among others.

**Conclusion:**

Currently, studies on network pharmacology are mainly associated with the exploration of action mechanisms of TCM. The main active ingredient in many Chinese medicines is quercetin. This ingredient may lead to deviation of research results, inability to truly analyze active ingredients, and even mislead the research direction of TCM. Such deviation may be because the database fails to reflect the content and composition changes of Chinese medicinal components. The database does not account for interactions among components, targets, and diseases, and it ignores the different pathological states of the disease. Therefore, network pharmacology should be improved from the databases and research methods.

## 1. Introduction

Network pharmacology expresses the relationship between drug components and their action targets in the form of networks. Through the analysis of network nodes, key active components and main drug targets can be obtained. Network pharmacology has improved the previous approach for exploring drug mechanisms, from the traditional single-target mechanism of the drug to the multitarget mechanism of drugs. Drugs often work by acting on multiple target proteins in the human body. It is obviously difficult to reveal the real action mechanism of drugs from the traditional single-target action mechanism research. Network pharmacology provides new methods and ideas for the exploration of mechanisms, research, and development of new drugs.

Due to the characteristics of TCM, complex compositions of prescriptions, and Chinese medicines, it is difficult to explain the mechanisms of TCM in disease treatment. Therefore, network pharmacology has been widely used in TCM. Studies on network pharmacology have explored the mechanisms of TCM, thereby providing information on its improvement. Various studies have used the TCMSP database to screen the components of compounds in Chinese medicines through OB (oral bioavailability) ≥30% and DL (drug-likeness)≥0.18 and then screen the active components and main action targets of Chinese medicines through degree, betweenness, and closeness centralities [1–3]. OB and DL mainly reflect whether the components contained in Chinese medicine can be absorbed and utilized by the human body. Degree centralities indicate the number of connections between a node and other nodes, closeness centralities indicate the average shortest path from one node to other nodes, and betweenness centralities indicate the number of times that a node serves as the shortest path between other nodes, which together reflect the importance of a node in the network. However, there is a need for further investigations on whether this research method can reflect the mechanisms of action of TCM.

In disease treatment using TCM, diseases should first be classified into different syndrome types according to their clinical manifestations. The different treatments, prescriptions, and drugs are proposed based on syndrome differentiations. Finally, drugs can only be applied to the human body through different decocting methods. Currently, these databases do not reflect the processes of TCM syndrome differentiation; therefore, it is challenging to meet the needs of TCM research. There is a need to sort out and analyze studies on network pharmacology. Scientometrics can well reveal the disciplinary development process, research status, and development trend and has been widely used in the research of various disciplines. Wang [4] explored the research trends of Chinese herbal medicine in relieving pain by applying scientometrics. Zhong [5] applied scientometrics to study the pathogenesis of epilepsy. Hancean [6] and Vasilyeva [7] explored the cooperative relationship between researchers through scientometrics. In this study, we explore the developmental course of network pharmacology and the relationship between network pharmacology and TCM through scientometrics. The findings will elucidate the challenges and inform about the need for improvement of network pharmacology.

## 2. Materials and Methods

### 2.1. Search Strategy

Literature data were obtained from the core collection of Web of Science, including the Science Citation Index Extended (SCI-Extended) and Emerging Sources Citation Index (ESCI) databases. The search term was “network pharmacology,” while the search date was from 1900 to July 1, 2021, (for SCI-EXPANDED database) and from 2015 to July 1, 2021 (ESCI database).

### 2.2. Hiscite Literature Analysis

History of cite (HisCite) is a citation map analysis software developed by Garfield. It is mainly used to show the relationships among different literature in a study field. The software can quickly find out the important literature and draw the developmental process of the field. In this study, the developmental process of network pharmacology was analyzed using the HisCite software.

### 2.3. CiteSpace Literature Analysis

CiteSpace is a scientific literature analysis tool that was jointly developed by Dr. Chaomei Chen from the School of Information Science and Technology of Drexel University and the WISE Laboratory of the Dalian University of Technology. The tool can be used to analyze the current research status of network pharmacology in countries and institutions and research topics and cited references of network pharmacology.

## 3. Results and Discussions

### 3.1. Literature Retrieval Outcomes

**Figure 1:**
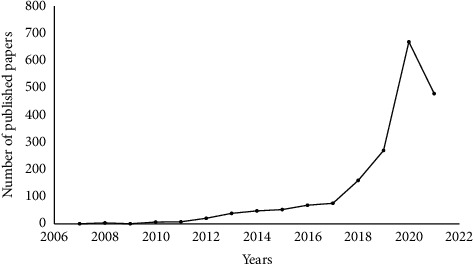
The literature on network pharmacology showed a rising trend from the date of database establishment to July 2021.

A total of 1905 articles were retrieved from the Web of Science database. The first study on network pharmacology was published in 2007. Since then, studies on network pharmacology have exhibited an increasing annual trend (Figure 1). Among the 1905 studies, 1634 were from China, 123 from the United States, 58 from India, and 41 from the United Kingdom. It was evident that China had the highest number of research publications on network pharmacology.

### 3.2. Hiscite Literature Analysis Results

**Figure 2:**
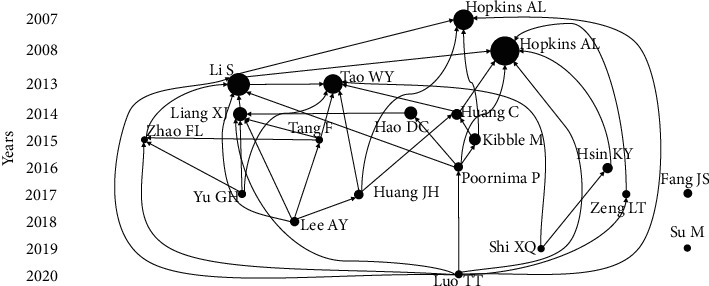
The top 20 works in literature on network pharmacology were screened out according to the LCS, and the citation relationship between them was sorted out according to the year of publication.

We used LCS (local citation score) in HisCite to select the top 20 articles for analysis of the development of network pharmacology. Local citation score (LCS) refers to the number of citations of a literature in all articles included in a study on network pharmacology, which therefore reflects the important position of the literature in the study. The citation times of the first four studies among the 20 selected studies were significantly higher than the rest (Figure 2).

The first study on network pharmacology (The initiator of network pharmacology) was published by Hopkins [8] in 2007. The publication plays an important role in this network because it elucidated the challenges of the single-target action mechanism of drugs and the complex network relationship between drugs and diseases. The study proposed using biological networks to assess relationships between drugs and targets, which can be considered the network pharmacology prototype. In 2008, Hopkins [9] proposed that network pharmacology could help in drug development and application, especially in the aspects of efficacy and adverse reactions. However, how to reasonably design the network pharmacology method to make it more consistent with the mechanisms of action of drugs is associated with various challenges. In 2013, network pharmacology attracted a wide range of attention in the TCM field. Tao [10], a Chinese researcher, constructed an application model of network pharmacology and used it to study active components of Chinese medicine prescription for the treatment of cardiovascular diseases. This study laid the foundation for future research on TCM using network pharmacology. In the same year, Li [11] noted that network pharmacology can not only be applied in studies on active ingredients in TCM but can also be used to explain the composition rules of TCM prescriptions and the relationship between TCM prescriptions and syndromes, thereby expanding the application scope of network pharmacology in the TCM field.

Network pharmacology plays an important role in exploring the mechanisms of Chinese herbal medicine in disease treatment [12, 13]. In terms of mechanisms of action of Chinese herbal medicines, Liang [14] used network pharmacology to study the action targets and pathways of Liuwei Dihuang Pills and deduced the diseases that the Pills could treat. Huang [15] explored the mechanisms of action of Yinchenhao Decoction and its relationship with different diseases. Further, Shi [16] explored the blood enrichment mechanisms of Danggui Buxue Decoction, among other drugs. Experts and scholars from different disciplines have evaluated the mechanisms of action of Chinese herbal medicines to treat various diseases. Regarding respiratory system diseases, Tang [17] explored the active components and targets of Mahuang Fuzi Xixin Decoction in the treatment of allergic rhinitis. Yu [18] explored the targets and pathways of Yinhuang Qingfei Capsule in the treatment of chronic bronchitis and molecular docking verification was also performed on the results. In metabolic diseases, Zhao [19] investigated the mechanisms of Simiao Pill in gout treatment, while Lee [20] explored the active components and targets of Yijin Decoction in the treatment of hyperlipidemia and atherosclerosis. Regarding encephalopathy, Huang [21] used network pharmacology to explore the applications of Chinese herbal medicines in depression treatment. Elsewhere, Fang [22] evaluated the mechanisms of action of Chinese herbal medicine to treat Alzheimer's disease. In cancer, Poornima [23] noted that network pharmacology was crucial for the development of anticancer drugs and the exploration of the potential of Chinese herbal medicines, such as Radix Acanthopanacis Senticosi, Radix Rhodiolae, and Fructus Schisandrae Chinensis for cancer treatment. Zeng [24] used network pharmacology to investigate the mechanisms of Yanghe Decoction in the treatment of HER2-positive breast cancer. We found that only one of the 20 works of the literature explored the action targets and biological mechanisms of non-Chinese herbal medicine (vitamin C) in liver injury treatment [25].

The molecular docking web server (SystemsDock) launched by Hsin [26] in 2016 can evaluate the relationship between protein and ligands in a more convenient and effective manner; therefore, it should be coupled to network pharmacology. However, the limitations associated with network pharmacology should be investigated further. In 2020, Luo [27] reported that network pharmacology plays an important role in target prediction, screening of bioactive compounds, and mechanism research of Chinese medicines. The discovery of new targets and drug mechanism research by network pharmacology is still in the qualitative stages; thus, the limitation of the dose-effect relationship between drugs and diseases cannot be solved.

### 3.3. Citespace Literature Analysis

#### 3.3.1. Literature Screening Results

**Figure 3:**
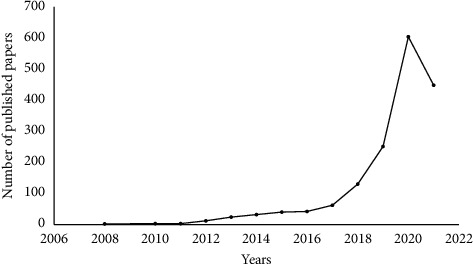
After excluding nonarticle type literature, articles on network pharmacology showed an increasing trend year by year from the date of database establishment to July 2021.

First, the literature was deduplicated and the document type classified as “article” screened, which could better reflect the current research hotspots and trends. A total of 252 non-“article” papers were deleted, including 9 corrections, 19 editorial materials, 3 letters, 34 meeting abstracts, 1 retraction, and 186 reviews, and finally, a total of 1653 articles were obtained for the current study. There was an annual increase in the number of articles on network pharmacology (Figure 3). The total number of articles published in 2021 was lower because the search date for the current study was up to July 1, 2021. Further, the number of articles published from 2008 to 2013 was small; therefore, only articles published between 2014 and 2021 were analyzed in the current study.

#### 3.3.2. Co-Countries Analysis

**Figure 4:**
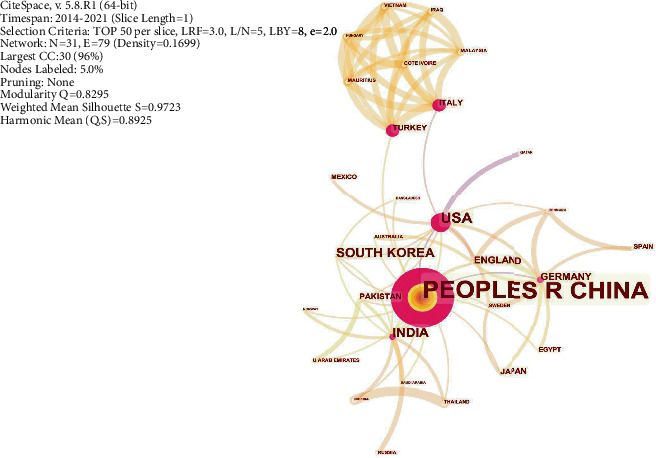
Co-countries analysis: China is the country with the most published articles on network pharmacology and occupies an important position in the international cooperation network.

China had the highest number of published studies on network pharmacology, followed by the United States. The size of the orange circle was found to be positively correlated with the number of studies (Figure 4), whereas the size of the purple circle was positively correlated with betweenness centrality. Countries with the highest betweenness centrality were China (0.93), the United States (0.57), Turkey (0.38), and Italy (0.25). These countries occupy an important position in the international cooperation network. The connecting lines between the nodes in Figure 4 indicate the cooperative relationship between countries. China had cooperating relations with 17 other countries, although the cooperation was not close among countries. The United States had cooperative relations with 12 countries whereby there was established close cooperation between the US and Qatar. Further, some countries, such as Turkey and Italy, exhibited closer cooperation.

#### 3.3.3. Co-Institutions Analysis

**Figure 5:**
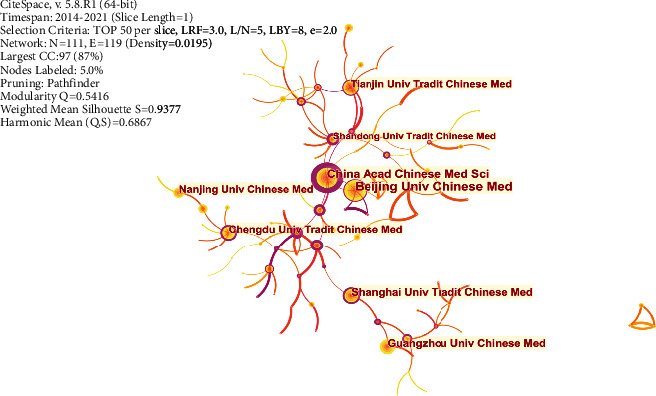
Co-institutions analysis: Beijing University of Chinese Medicine is the organization that publishes the most articles on network pharmacology, but the China Academy of Chinese Medical Sciences is more important in the institutional cooperation network.

According to the results of the present study, institutions with the highest number of published articles were Beijing University of Chinese Medicine, followed by China Academy of Chinese Medical Sciences, Shanghai University of Chinese Medicine, Guangzhou University of Chinese Medicine, Chengdu University of Chinese Medicine, Tianjin University of Chinese Medicine, and Nanjing University of Chinese Medicine (Figure 5). Further, it was also evident that the institutions were mainly concentrated in the Chinese medicine colleges and universities in China, and this was an indication that the research of network pharmacology is mainly in the field of Chinese medicine. However, it was noted that there was no close relationship in cooperation between the studied institutions. In terms of betweenness centrality, the value of the China Academy of Chinese Medical Sciences was found to be 1.05, whereas that of the Beijing University of Chinese Medicine was 0.15. This was an indication that the China Academy of Chinese Medical Sciences occupies a more crucial position in the cooperation network of various institutions.

#### 3.3.4. Co-Keywords Analysis

**Figure 6:**
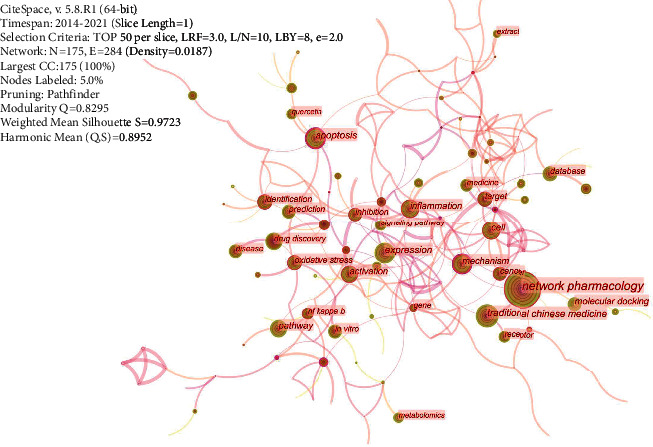
Co-keywords analysis: The most widely studied topic in network pharmacology is TCM. Network pharmacology is mainly used to explore the action mechanism of TCM.

**Figure 7:**
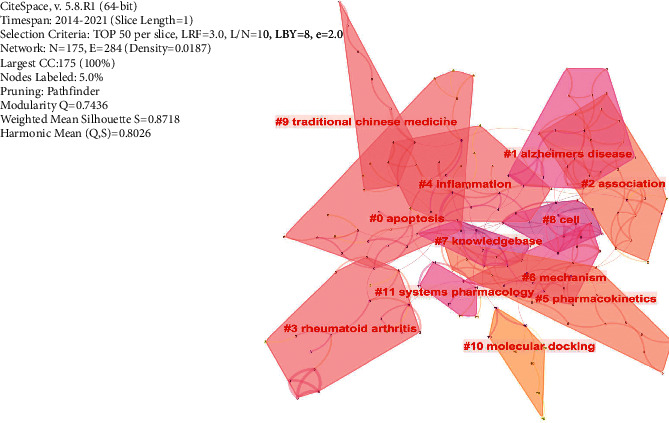
Keywords clustering analysis: Clustering analysis showed that the topics of network pharmacology research mainly revolved around traditional Chinese medicine, diseases, and mechanisms.

**Figure 8:**
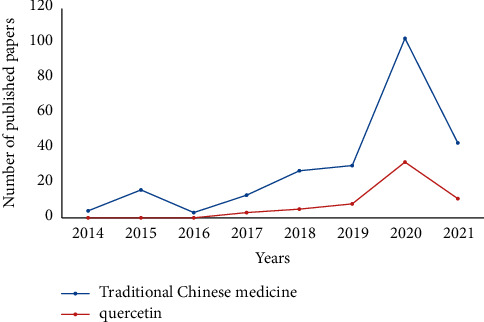
The keywords of Traditional Chinese medicine and quercetin are increasing year by year, and network pharmacology has been increasingly studied in this field.

The keywords with the same meaning were first combined in the present study. The keywords with higher research frequency included network pharmacology, Traditional Chinese Medicine, mechanism, pathway, and target (Figure 6). The analysis of the keywords clustering for the present study was as shown in Figure 7. Cluster labels were selected using index items and then expressed using the log-likelihood ratio. Results of the current study showed the clustering information of *Q* = 0.7436 and *S* = 0.8718, which indicates that the clustering was good (Modularity: clustering module value (*Q* value), and it was generally assumed that *Q* > 0.3 indicated that the clustering structure was significant. According to Silhouette, clustering average contour value (S value), is generally considered that clustering with *S* > 0.5 is reasonable and *S* > 0.7 means that the clustering is convincing). It was evident from the figure that the relationship between the clusters was relatively close. Further, the keywords frequency and clustering analysis showed that the research fields of network pharmacology were mainly related to the mechanisms of action for TCM in treating diseases. The changing trends of some keywords within the year are indicated, as shown in Figure 8. Further, it was found that the annual application of network pharmacology to the study of TCM had a rising trend. Quercetin was the most widely studied component in Chinese medicines, and research on this compound also showed an increasing trend annually.

It was found that a total of 59 articles were published on quercetin. In the present study, the first eight articles with high citation frequency and covering different diseases and prescriptions are analyzed, as shown in Table 1. For instance, in the treatment of asthma, Song [29] explored the mechanism of action for Maxing Shigan Decoction for the treatment of asthma. Through network analysis, it was also found that quercetin and kaempferol acted on a total of 172 and 106 targets, respectively. Therefore, the two may be the key bioactive components of Maxing Shigan Decoction. In the treatment of COVID-19, Wang [30] conducted a study through network pharmacology and concluded that both quercetin and kaempferol in Maxing Shigan Decoction can reduce fever symptoms by inhibiting the expressions of interleukin-1*β*, interleukin-6, and tumor necrosis factor-*α* to treat COVID-19. Based on network pharmacology analysis, another study conducted by bu Tao [32] also concluded that baicalein and quercetin in the Huashi Paidu formula may play a role in the treatment of COVID-19 through the regulation of multiple signaling pathways using ACE2. In addition, Qin [33] noted that quercetin was a common compound in astragalus and safflower with the highest degree of centrality in the treatment of chronic kidney disease (CKD). This indicated that quercetin interacted with the largest number of targets; hence, it may play a key role in the network. Therefore, it was evident that quercetin may exert its potential biological effects through multitarget in treating CKD. Elsewhere, through topology analysis, according to the degree centrality, and betweenness centrality, Zeng [31] speculated that quercetin, *β*-sitosterol, and kaempferol were the important active compounds, which played a key role in the treatment of Alzheimer's disease using Chaihu Shugan San. Further, in the treatment of polycystic ovary syndrome (PCOS) through network pharmacology, Liu [34] reported that quercetin (153 targets) and kaempferol (63 targets) were the main active components of Erxian Decoction. According to a study conducted by Mao [28], the treatment of cancer through network pharmacology analysis, it was reported that most of the 10 Chinese medicines for the treatment of metastatic breast cancer (*Cervus nippon* Temminck, Ginger Charcoal, Citri Reticulatae Viride Pericarpium, Phytolaccae Radix, Licorice, *Trichosanthes kirilowii* Maxim, Citri Reticulatae Folium, *Panax notoginseng*, Epimedium Herb, and Fritillariae Thunbergii Bulbus) contained quercetin and kaempferol. Therefore, quercetin and kaempferol may play a major role in the treatment of metastatic breast cancer. According to a study conducted by Huang [15], it was found (through network pharmacology) that Yinchenhao Decoction can treat cancer, cardiovascular, and immune diseases, whereby quercetin, *β*-sitosterol, and isorhamnetin may be the main active components.

Although network pharmacology plays an important role in the research of TCM, analysis of the available studies also noted its various disadvantages. It was evident that network pharmacology (Maxing Shigan Decoction, Yinchenhao Decoction, Chaihu Shugan San, Erxian Decoction, or Shenkang) has different components and also treats different diseases (cancer, asthma, COVID-19, Alzheimer's disease, chronic kidney disease, and polycystic ovary syndrome). However, the results of the final analysis in the current study showed that the main active components were related to quercetin, and such development will inevitably affect the development of TCM. Quercetin is a common chemical substance found in 188 Chinese medicinal materials, which has several important effects. However, there is a need for deep consideration of the fact that quercetin is the active component in treating diseases in most prescriptions. Furthermore, the reason why quercetin is the main active component can be analyzed from the network pharmacology methods.

(1) *The Component Contents of Chinese Medicines*. Currently, the common Chinese herbal medicines that target databases such as the TCMSP database do not take into account the content of each component of the herbal medicine or dose of the herbal medicine in prescription, although they have the components and targets of common herbal medicines and the OB and drug similarity. A certain blood concentration is required for the drug to take effect. Western drugs such as phenobarbital do not produce any effect when their dose is below the threshold. However, with an increase in dose, it will successively produce the effects of sedation, hypnosis, anticonvulsant, and antiepileptic and also lead to the occurrence of central nervous paralysis and death of patients in serious cases [35]. Chinese medicines emphasize proper dosage because different doses will have different effects. For instance, with different doses of Radix Bupleuri, the effects greatly varied, from 1 to 3g to lift the yang qi, 9 to 15g to soothe the liver and relieve depression, and more than 15g to relieve exterior syndrome and fever [36]. If a certain herbal medicine contains few components of quercetin that cannot reach the concentration in blood, it will not have the corresponding effect. A separate study previously showed that there is a correlation between the efficacy and dose of quercetin. For instance, it has been reported that quercetin can protect rat DNA from oxidative damage induced by ferric sulfate at a low dose (500 mg/kg), whereas a high dose will produce cytotoxicity [37].

(2) *Composition Changes of Chinese Medicines*. It has been reported that decocting time and temperature can affect the components and efficacy of Chinese medicines. For example, a short decocting time (15 minutes) was found to be conducive to decocting bisanthrone glycosides and anthraquinone glycosides of Radix et Rhizoma Rhei, mainly to exert the effect of diarrhea. On the other hand, a long decocting time (60 minutes) was found to be conducive to the decocting of tannins of Radix et Rhizoma Rhei, with heat-clearing and detoxicating as the main effect [38]. After decocting for 5 minutes, it was noted that the volatile oil content of aromatic drugs was the highest, and it was significantly decreased with the prolonged decocting time [39]. In addition, it could be seen that the decocting process would cause changes in the functions and compositions of drugs. However, the databases currently used in network pharmacology studies have not considered these issues. Therefore, the components of individual herbs were only retrieved from the databases without considering the changes in the compositions of the drugs caused by decocting.

*(3) Chinese Medicines Components and Targets Number.* Network pharmacology research mainly relies on the degree, betweenness, and closeness centralities to determine the active component of a drug. Through the TSMSP database, a total of 154 targets of quercetin were obtained, whereas each poriferast-5-en-3beta-ol and Pyrethrin II corresponded to two targets only. It was evident that the number of targets corresponding to quercetin far exceeded that of the other components and easily occupied an important position in the network analysis. Therefore, as long as the components of Chinese medicine contain quercetin, it can be easily analyzed according to important active components. The number of targets corresponding to the common active component kaempferol in the previous studies was relatively large at 62. Further, it was found that there was no absolute correspondence between the number of targets and their efficacy. For example, only three targets of metformin were retrieved from the DrugBank database. However, metformin is still the first-line drug for treating type 2 diabetes mellitus [40]. Furthermore, if metformin is analyzed by network pharmacology, it is difficult to identify metformin as an important active ingredient due to the small number of corresponding targets. It was evident that there was a deviation in judging the active components of a drug by the number of targets and betweenness centrality only.

*(4) The Relationship between Components and Targets*. In the current network pharmacology studies, it was found that the strong effects of components on targets were not considered. For instance, codeine, pethidine, and methadone can act on *μ* receptors. However, the acting strengths of the three are different, methadone > pethidine > codeine [41]. Furthermore, the relationship between the components and targets in network pharmacology analysis was regarded as a straight line, and the action intensity of this straight line was not considered.

*(5) The Relationship between Targets and Diseases*. The effect of targets on the disease was also not considered in the current analysis of network pharmacology. For instance, morphine can exert analgesic effects by affecting multiple receptors, including *μ*, *δ*, and K. The main receptor for analgesic effects of morphine is *μ*, whereas *δ* receptor-mediated analgesic effects are not significant. Therefore, it is evident that the *μ* receptor has a closer relationship with analgesia [41]. Although the number of corresponding targets of a component is small, it has been found that if the target plays an important role in the disease, the component is still an important active component.

*(6) Different Syndromes of Diseases*. Traditional Chinese medicine (TCM) believes that due to different constitutions, people suffer from the same disease with different clinical manifestations. Therefore, patients can use different herbs to treat the same disease. For instance, in the Guideline for the prevention and treatment of type 2 diabetes mellitus in China (2020 edition), type 2 diabetes mellitus is divided into the deficiency of both qi and yin, damp-heat in the intestinal tract, and liver and stomach stagnation heat [40], which is a common phenomenon in clinical practice. Therefore, there should be some connection between diseases and syndromes. The current databases only match corresponding targets according to diseases, and there is no database for corresponding targets for different syndromes of the diseases. Most Chinese medicinal prescriptions have their own adaptive syndrome types; hence, the current databases cannot meet the requirements of the studies.

#### 3.3.5. Co-Cited References Analysis

**Figure 9:**
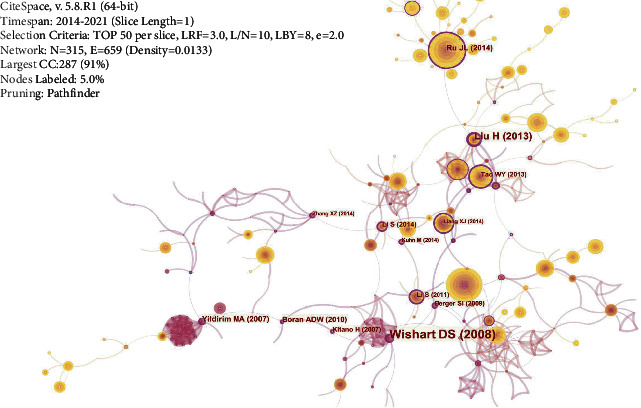
Co-cited references analysis: the size of the orange circle indicated how many times the article had been cited, and the size of the purple circle indicated the importance of the article in the network.

Results of the present study found that the size of the purple circle was positively correlated with the betweenness centrality, which reflects the important role of literature in the development of network pharmacology (Figure 9). The first 10 articles with the highest betweenness centrality were mainly related to database development and methodological innovation. Furthermore, they were found to play a positive role in promoting the development of network pharmacology. In the research and development of the database, the DrugBank database had been widely used for drug target discovery and drug interaction prediction since its release in 2006 and hence facilitated the research of network pharmacology [42]. Aware of the complexity of Chinese herbal medicine components, in 2014, a study conducted by Ru [43] developed the TCMSP database to promote the development of Chinese herbal medicine. This database provided data on OB, drug similarity, compounds, and targets for the screening and evaluation of drug components, which helped reveal the mechanism of action of Chinese medicine.

In terms of methodological innovation, Kitano, 2007 [44], noted that there were limitations in analyzing the efficacy of drugs for the treatment of diseases solely from the aspects of drug targets and mechanisms of action. It has been found that the body has robustness, which can interact with drugs to affect the expected efficacy. Therefore, the robustness of the body should be taken into account in drug development. Elsewhere, Yildirim [45] used DrugBank and OMIM databases to explore the relationship among drugs, targets, and diseases by constructing a protein-protein interaction network, providing ideas for the development of network pharmacology. In 2009, Berger [46] summarized system pharmacology and reported that the pharmacology system can identify drug targets and mechanisms of action by network analysis. Therefore, the studies provided assistance for drug research and development and application. In 2010, Boran [47] pointed out that system biology can explore the mechanisms of action and adverse reactions of drugs through multitargets and was suitable for developing new drugs. In 2011, Li [48] found that drugs were usually composed of multiple components, and the relationship between each component was relatively complex. Therefore, so he created NIMS (Network target-based Identification of Multicomponent Synergy) to explore how to screen the components of drug synergy to increase the efficacy of the drugs. In 2013, Tao [10] constructed the application model of TCM in network pharmacology, and the article laid a foundation for future research on TCM using network pharmacology. In the same year (2013), Liu [49] also created a network pharmacology analysis model integrating OB, drug similarity, target recognition, and network analysis with Licorice as an example. Elsewhere, Li [50] analyzed the literature on network pharmacology and proposed that network pharmacology is an emerging field combining TCM with modern drug research, development, and treatment, which would help to find the complex mechanism of action of the Chinese medicine for the treatment of diseases.

## 4. Conclusion

It is evident that since its discovery by Hopkins in 2007, network pharmacology has significantly improved. Chinese scholars have extensively published literature on network pharmacology, followed by scholars from the United States of America. Further, it was found that research conducted in China provides a significant international cooperation network; however, its cooperation relationship with other countries is not high. In addition, the correlation between the United States and Qatar cooperation is relatively high. Chinese medical colleges and universities play an important role in publishing articles on network pharmacology. Notably, it was found that the Beijing University of Chinese Medicine has the largest number of published articles, followed by the Chinese Academy of Chinese Medical Sciences. However, it was evident that the institutions are not significantly associated.

Network pharmacology research mainly explores the mechanism of action of TCM in the treatment of diseases, thus providing significant findings on the efficacy of TCM. Although several articles on network pharmacology have been published, the studies are characterized by some shortcomings. Quercetin comprises key active components of Chinese medicine prescriptions. In addition, the efficacy of the active components has been explored using network pharmacology approaches. Notably, network pharmacology does not consider the dose-effect relationship of drugs and changes of components of Chinese medicines in the decocting process. Moreover, it was noted that the studied approaches do not determine the intensity of interaction between drugs and targets or between targets and diseases. It has been found that the current studies have reported compounds with many targets as potential active components. A direct disadvantage of this method is that similar active components of different prescriptions are identified, whereas compounds with fewer targets are ignored. Therefore, the results of network pharmacology analysis may not be accurate, and thus the real active components are not analyzed. This results in the waste of high levels of human resources and material resources and delays in the development of TCM.

Therefore, improvement and development of databases and methodological innovation should be conducted to alleviate the noted shortcomings of the network pharmacology approach. The database of Chinese medicine should be further enriched to study the relationship between Chinese medicines doses and targets, and this will alleviate the shortcoming of different therapeutic effects at different doses. Effects of Chinese medicines doses on the targets and therapeutic effects should also be explored in the future. In addition, several Chinese medicines can only be applied to the human body after being decocted. Different decocting methods affect the efficacy of Chinese medicines. Therefore, the effects of decocting methods on the content of drug components, efficacy, targets, and mechanisms should be investigated.

Further, novel approaches should be developed to solve the problem of the number of targets corresponding to different compound components. The main active components of drugs cannot only be judged by the degree centrality and betweenness centrality. A relatively fair comparison method should be developed to evaluate different compounds to achieve a relatively universal baseline of compound comparison. Moreover, the action intensity of components on targets should also be explored. Action intensity varies between components and targets; hence, weights should be assigned according to action intensity. The relationship between targets and diseases should be considered because different targets have different effects on diseases. To achieve accurate results, weight should be assigned according to the action intensity. Further studies should explore whether the corresponding targets are different for different syndromes of the same disease, thus promoting research and development of western medicine and providing information on the mechanism of action of TCM. A database should be constructed and new methods explored to promote the development of network pharmacology. In conclusion, it is evident that network pharmacology has provided novel ideas and also significantly improved research on TCM. However, the rationality of the results should be ensured and research on network pharmacology should include the mechanism of action of TCM. Therefore, the advances will improve the role of network pharmacology in the development of TCM.

## Figures and Tables

**Table 1:** There were as many as 59 articles on quercetin in network pharmacology, and the top 8 articles with the highest citation frequency were analyzed.

Citations	Fist author	Year	Journal
48	Huang JH [15]	2017	Molecular Medicine Reports
**35**	Mao Y [28]	2017	Oncotarget
**26**	Song WJ [29]	2018	Scientific Reports
**17**	Wang YX [30]	2020	European Review for Medical and Pharmacological Sciences
**14**	Zeng *Q* [31]	2019	Biomedicine and Pharmacotherapy
**13**	Tao QY [32]	2020	Drug Development and Industrial Pharmacy
**11**	Qin TY [33]	2020	Journal of Ethnopharmacology

## Data Availability

Original data presented in the study are included in the article. Further inquiries can be directed to the corresponding authors.

## Supplementary Materials

Supplementary Materials The supplementary materials were the basic information of 1905 articles downloaded from the Web of Science database in this study, including keywords, references, and the authors countries and institutions.
